# Fully Spray-Coated Triple-Cation Perovskite Solar Cells

**DOI:** 10.1038/s41598-020-63674-5

**Published:** 2020-04-20

**Authors:** James E. Bishop, Connor D. Read, Joel A. Smith, Thomas J. Routledge, David G. Lidzey

**Affiliations:** Department of Physics & Astronomy, University of Sheffield, Hicks Building, Hounsfield Road, Sheffield, S3 7RH UK

**Keywords:** Solar cells, Renewable energy

## Abstract

We use ultrasonic spray-coating to sequentially deposit thin films of tin oxide, a triple-cation perovskite and spiro-OMeTAD, allowing us fabricate perovskite solar cells (PSCs) with a champion reverse scan power conversion efficiency (PCE) of 19.4% on small-area substrates. We show that the use of spray-deposition permits us to rapidly (>80 mm s^−1^) coat 25 mm × 75 mm substrates that were divided into a series of devices each with an active area of 15.4 mm^2^, yielding an average PCE of 10.3% and a peak PCE of 16.3%. By connecting seven 15.4 mm^2^ devices in parallel on a single substrate, we create a device having an effective active area of 1.08 cm^2^ and a PCE of 12.7%. This work demonstrates the possibility for spray-coating to fabricate high efficiency and low-cost perovskite solar cells at speed.

## Introduction

Since the initial reports of perovskite solar cells (PSCs) in 2009 the power conversion efficiencies (PCEs) of such devices have risen from 3.8%^1^ to 25.2%^2^. Perovskites have many properties which make them attractive materials for solar cell applications, including efficient light absorption, tuneable band gap, long charge-carrier lifetimes and high defect tolerance^3–8^. However it is the relative ease by which perovskite films can be deposited from solution that has generated the greatest interest, as this potentially allows high volume manufacture of photovoltaic modules at low cost and low temperature. This could allow a dramatic reduction in the energy payback time of a commercial module to less than half a year^9^. In order for this to become a reality, it is necessary to demonstrate that perovskite solar cells can be fabricated using industrially compatible coating techniques.

Currently most perovskite device optimisation is performed using spin coating; a simple and reliable technique capable of producing highly uniform thin films. However spin coating is usually only suitable for coating small substrates on the order of square centimetres, rather than square meters^10^. Spin coating is also a wasteful coating process, as the majority of the ink to be coated is thrown from the substrate during deposition. Consequently, increased research effort is being focused towards scalable deposition techniques such as blade-coating^11^, slot-die coating^12^, inkjet printing^13^, and spray-coating^14^.

In this paper, we report on the deposition of perovskite PV devices via ultrasonic spray-coating and their partial scale-up. Spray-coating is an attractive technique for high volume manufacturing, as it allows large areas to be coated at high-speed with only minimal loss of coating ink. In contrast to regular aperture based spray-coaters, ultrasonic spray-coaters utilise piezoelectric transducers to shear the solution to be deposited into a mist of micron sized droplets characterised by a smaller average size than those produced by a conventional air-brush device^15^. This, in principle, allows the deposition of more uniform coatings. The first spray-coated perovskite solar cells fabricated via ultrasonic spray-coating deposited a perovskite from a 3:1 mixture of methylammonium iodide and lead chloride and achieved an average PCE of 7.8%^14^. Here, a simple single-pass deposition technique was used in which the spray-head moved across the substrate creating a “wet film”, which – after thermal annealing – formed a CH_3_NH_3_PbI_3−x_Cl_x_ perovskite capable of reaching a peak efficiency of 11% when integrated into a device^14^. Following this, a series of approaches have been explored to improve film uniformity and device performance. These include the use of two-step deposition protocols^16^, continuous soaking of the substrate^17^, anti-solvent bath treatments^18^, the use of multiple spray-passes^19^, low vacuum treatments^20^, megasonic spray-coating^21^, and hot-air treatments^22^. Indeed, many groups can now reliably produce spray-coated PSCs with an average PCE in the mid-teens with the best devices reaching a reverse scan PCE of 18.5% and a stabilised PCE of 17.3%^23^. Furthermore spray deposition has been utilised to probe compositional space in mixed cation systems by controlling the flow of two inks delivered to the spray head prior to atomisation^24,25^. The interested reader is directed to a recent review that charts the development of spray-coated perovskite PV^10^.

It is important to emphasize that the majority of papers on spray-coated PSCs rely on spin-coating to deposit the electron and hole transport layers that are used to extract charges from the active perovskite layer^16–23,26,27^. Ideally however, spray-coating should be used to deposit all solution processable layers within a PSC as this would replicate a practical manufacture process. In previous work, we demonstrated a fully spray-cast perovskite device based on an ‘inverted’ (*p-i-n*) architecture where the PEDOT:PSS, CH_3_NH_3_PbI_3−x_Cl_x_ perovskite and PCBM were deposited by spray-coating, with devices reported having an average PCE of 7.1%^28^. By switching to an *n-i-p* architecture and sequentially spray-coating compact TiO_2_, mesoporous TiO_2_, CH_3_NH_3_PbI_3−x_Cl_x_ perovskite and spiro-OMeTAD, we have also been able to create PV devices yielding an average PCE of 9.2%^29^.

Recently perovskite solar cells having a PCE of 20% have been demonstrated in which all solution processed layers (namely SnO_2_, perovskite, and spiro-OMeTAD) were deposited via air-blading^30^. This arguably represents the state-of-the-art for scalable perovskite deposition methods. In this article we perform a similar study, building upon our previous work to spray-coat all solution processable layers within a device, and incorporate a low vacuum treatment step to crystallise a “triple-cation” perovskite layer. We then scale up our fabrication process to larger area substrates to fabricate efficient PSCs. We believe that this represents an important proof-of-concept that could be transferable to an industrial manufacturing environment.

## Results

### Device fabrication

We have used a Prism Ultra-coat 300 system (Ultrasonic Systems Inc.) ultrasonic spray-coater operated in low humidity air to deposit the nanoparticle tin oxide (np-SnO_2_) and spiro-OMeTAD layers. The perovskite layer was instead deposited using a Sonotek Exactacoat equipped with an “Impact” spray-head that was located inside a nitrogen-filled glovebox. Here, the inert environment was critical to allow us to control the drying and crystallisation dynamics of the perovskite, removing the effect of oxygen and moisture. Both spray-coaters were equipped with a motorised gantry that allowed the spray-head to be scanned across a substrate in a controlled manner. All spray-coating described here is based on a simple “single pass” deposition process in which the spray-head was moved over the substrate in a straight line at a range of coating velocities (80 to 180 mm s^−1^) depending on the particular layer being coated. This allowed a roll-to-roll industrial coating process to be simulated, in which a sheet is continuously fed through a coating system. We have found that by controlling head height, the relative velocity of the head as it passes across the surface and the fluid flow rate, we can control the thickness of the resultant layer. We have found that it is also necessary to maintain the substrate at an elevated temperature during deposition to control both surface wetting and the subsequent drying of the “wet film”^15,31^.

**Figure 1:**
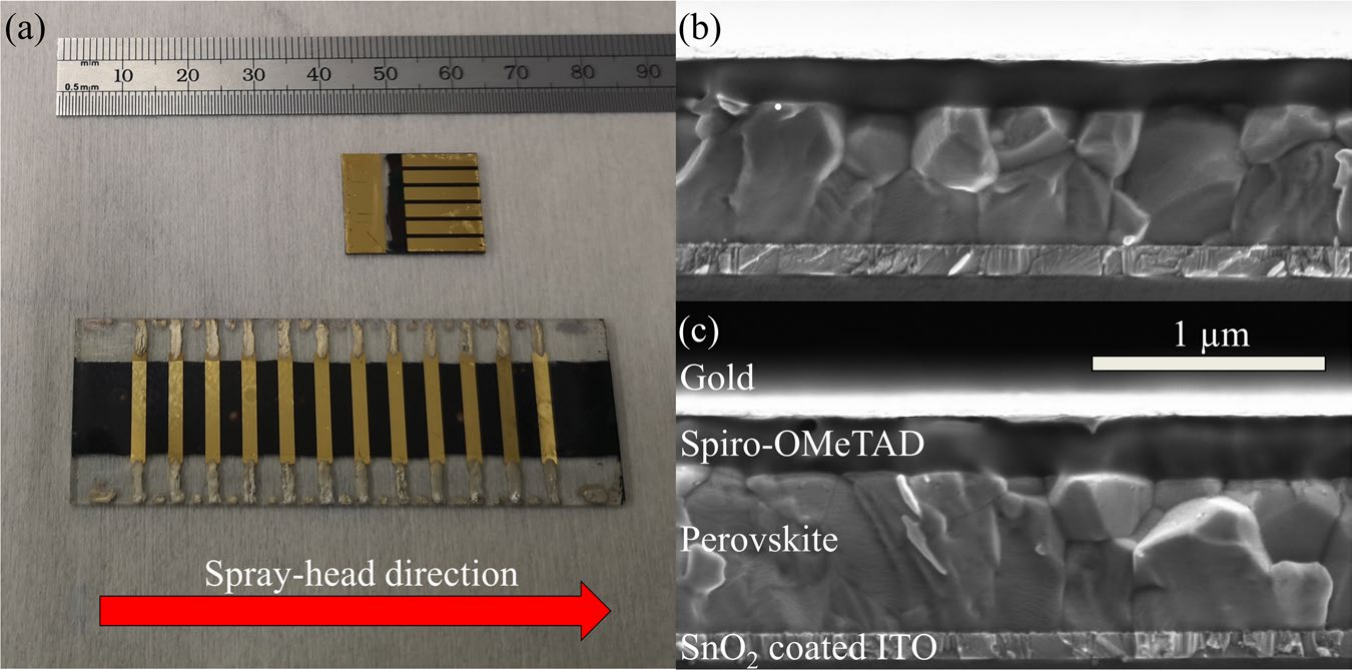
Part (**a**) shows a photograph of small and large-area fully spray-coated perovskite solar cells. Parts (**b**,**c**) show a cross-sectional SEM image of complete devices incorporating a spray-cast perovskite layer. The device in part (**b**) utilises spin cast SnO_2_ and spiro-OMeTAD layers whereas the device in part (**c**) is fully spray-coated.

Figure 1(a) shows images of small and large-area spray-coated devices. Small-area devices were fabricated on 15 × 20 mm ITO substrates that were patterned into six 2 × 2 mm pixels. These pixels were characterised through an illumination mask having an aperture of 2.5 mm^2^. Larger-area devices were also fabricated on 25 × 75 mm ITO substrates, and were patterned into twelve 10 × 2 mm pixels. An image of such devices is also shown in Fig. 1(a), with the red arrow indicating the direction that the spray-head moved across the substrate. These pixels were then characterised through an illumination mask having an aperture of 15.4 mm^2^.

The devices fabricated were based on the following planar architecture: ITO/np-SnO_2_/perovskite/spiro-OMeTAD/Au. Tin oxide layers were deposited from a commercially available nanoparticle dispersion^32^ diluted in water, which we have both spin and spray-coated^33^. After deposition, the films were annealed for 30 minutes at 150 °C before being subject to a 15 minute UV ozone treatment. Films were then transferred to a nitrogen glovebox for deposition of the perovskite layer using a spray-coating process that involves an exposure to a vacuum (so-called vacuum-flash assisted solution processing (VASP))^20,34^. Here the perovskite precursor was based on a stoichiometric mixture having the composition Cs_0.05_FA_0.81_MA_0.14_PbI_2.55_Br_0.45_, dissolved in a 4:1 mixture of DMF:DMSO. After the substrate was coated with the precursor ink, it was loaded into a sealed box that was then rapidly evacuated to a coarse vacuum (80 Pa) for 1 minute. This ensures high film quality by controlling nucleation of the perovskite phase. After this treatment, the substrate was removed from the vacuum and annealed at 120 °C for 20 minutes to fully crystallise the perovskite layer. Spiro-OMeTAD was either spin-coated onto the substrate in a glovebox environment or spray-coated in air using a process similar to one we reported previously^29^. Here the spray-cast ink had a lower concentration and was dissolved in a 1:1 mixture of chloroform and chlorobenzene to enhance surface wetting and accelerate film drying. Finally, thermal evaporation was used to deposit and pattern the gold top contact through a shadow mask. Further experimental details are provided in the methods section.

### Device characterisation

**Figure 2:**
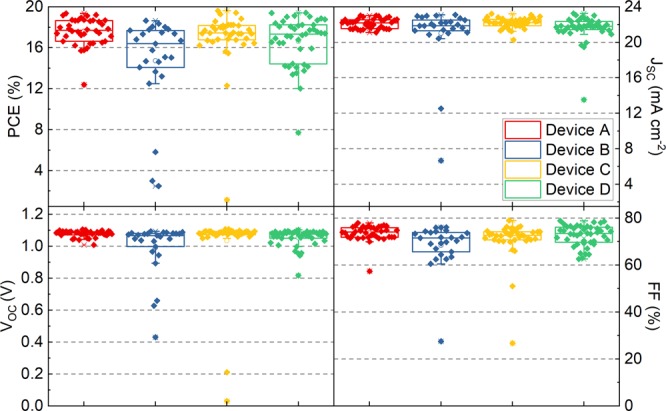
Box plots showing reverse scan PSC performance recorded from small-area (2.5 mm^2^) devices A-D (see Table 1 for a description of device labels).

We have fabricated a series of photovoltaic devices, in which the np-SnO_2_ and spiro-OMeTAD layers were either deposited via spin-coating or spray-coating in order to quantify the effect of the process route on device performance. In all cases, the perovskite layer was deposited via spray-coating. Table 1 lists the various devices fabricated, in which both transport layers were deposited via spin coating (defined as Device A), or by spray-coating (Devices D and E), or by some combination of spin- or spray-coating (Devices B and C). Figure 1(b) shows an SEM cross-section of a spray-cast perovskite solar cell where the transport layers were spin-coated (Device A). Figure 1(c) shows a similar cell in which all three layers were deposited via spray-coating (Device D). Over these length scales it is apparent that there is no significant morphological difference between either of the devices, with the thickness of all of the layers being relatively uniform. A summary of the reverse scan performance metrics of these devices tested under standard AM 1.5 illumination is presented in Table 1, together with box plots in Fig. 2.

**Table 1:** A summary of PSC performance metrics extracted from the reverse scan together with the deposition technique used to fabricate each layer. Data shown using a bold font are the values determined from the most efficient device with the average and standard deviation presented in parenthesis.

Device	Device A	Device B	Device C	Device D	Device E
Area (mm^2^)	2.5	2.5	2.5	2.5	15.4
*np-SnO*_2_	Spin	Spray	Spin	Spray	Spray
*Perovskite*	Spray	Spray	Spray	Spray	Spray
*Spiro-OMeTAD*	Spin	Spin	Spray	Spray	Spray
PCE (%)	**19.4** (17.6 ± 1.3)	**18.6** (14.7 ± 4.4)	**19.6** (17.0 ± 2.9)	**19.4** (16.6 ± 2.4)	**16.3** (10.3 ± 4.0)
J_SC_(mA/cm^2^)	**22.5** (22.1 ± 0.5)	**22.5** (21.0 ± 3.5)	**23.2** (22.2 ± 0.6)	**23.1** (21.6 ± 1.5)	**23.8** (20.0 ± 2.2)
V_oc_ (V)	**1.11** (1.08 ± 0.02)	**1.09** (0.99 ± 0.17)	**1.11** (1.03 ± 0.21)	**1.09** (1.05 ± 0.05)	**1.12** (0.96 ± 0.21)
FF (%)	**78** (74 ± 3)	**76** (68 ± 10)	**76** (71 ± 8)	**77** (73 ± 4)	**61** (50 ± 11)
Failed Devices	2/40	3/28	2/40	1/44	3/48

It can be seen that no drop in performance in the small-area devices is observed as more layers are spray-cast. In Device A the np-SnO_2_ and spiro-OMeTAD layers were spin-coated while the (VASP treated) perovskite layer was spray-cast, with devices having a peak PCE of 19.4% with an average PCE of 17.6 ± 1.3%. To the best of our knowledge this is the highest reported peak efficiency for a PSC containing a spray-coated perovskite layer. Importantly we have optimised our previous deposition protocol^20^ and find that the vacuum exposure time can be shortened from 5 minutes to 1 minute whilst yielding improved PV performance (see Fig. S1).

We find that if the np-SnO_2_ layer is spray-cast (Device B) there is a slight reduction in average PCE to 14.7 ± 4.4% that we attribute to a low-level of defects introduced into the film during spray-deposition. We suspect that such defects occur as the np-SnO_2_ layer is very thin, and thus if its surface becomes contaminated by any dust or debris, it can cause dewetting of the perovskite layer, creating voids that act to reduce device performance (see Fig. S2). The magnitude of this effect varies from sample to sample and can cause a large drop in both V_OC_ and FF (see Fig. S3). We classify devices having less than 1% PCE as “failed devices” with the number of such devices reported in Table 1. Note, the performance metrics of these devices have been omitted from our statistical analysis. If the deposition is performed in a cleaner environment then we anticipate no loss in performance when spraying this layer. For a more in depth analysis of spray-coated np-SnO_2_ films we direct the reader to other recent work in which we develop this process^33^.

On spray-coating the spiro-OMeTAD (Device C) we find that there is only minimal loss in device performance, with devices having an average PCE of 17.0 ± 2.9%. Here, we have carefully controlled the thickness of the spray-cast spiro-OMeTAD (≈200 nm) by adjusting the concentration of spray-solution so that it matches that of the spin-cast layer. Indeed, we have found that if the spiro-OMeTAD layer thickness is greater than this optimum value, it results in reduced performance (see Fig. S4) and increased hysteresis. As a result of this optimisation, when all three layers were spray-coated (Device D) we were able to produce PSCs with an average PCE of 16.6 ± 2.4% and a peak efficiency of 19.4%.

### Surface profilometry and laser-beam-induced current mapping

**Figure 3:**
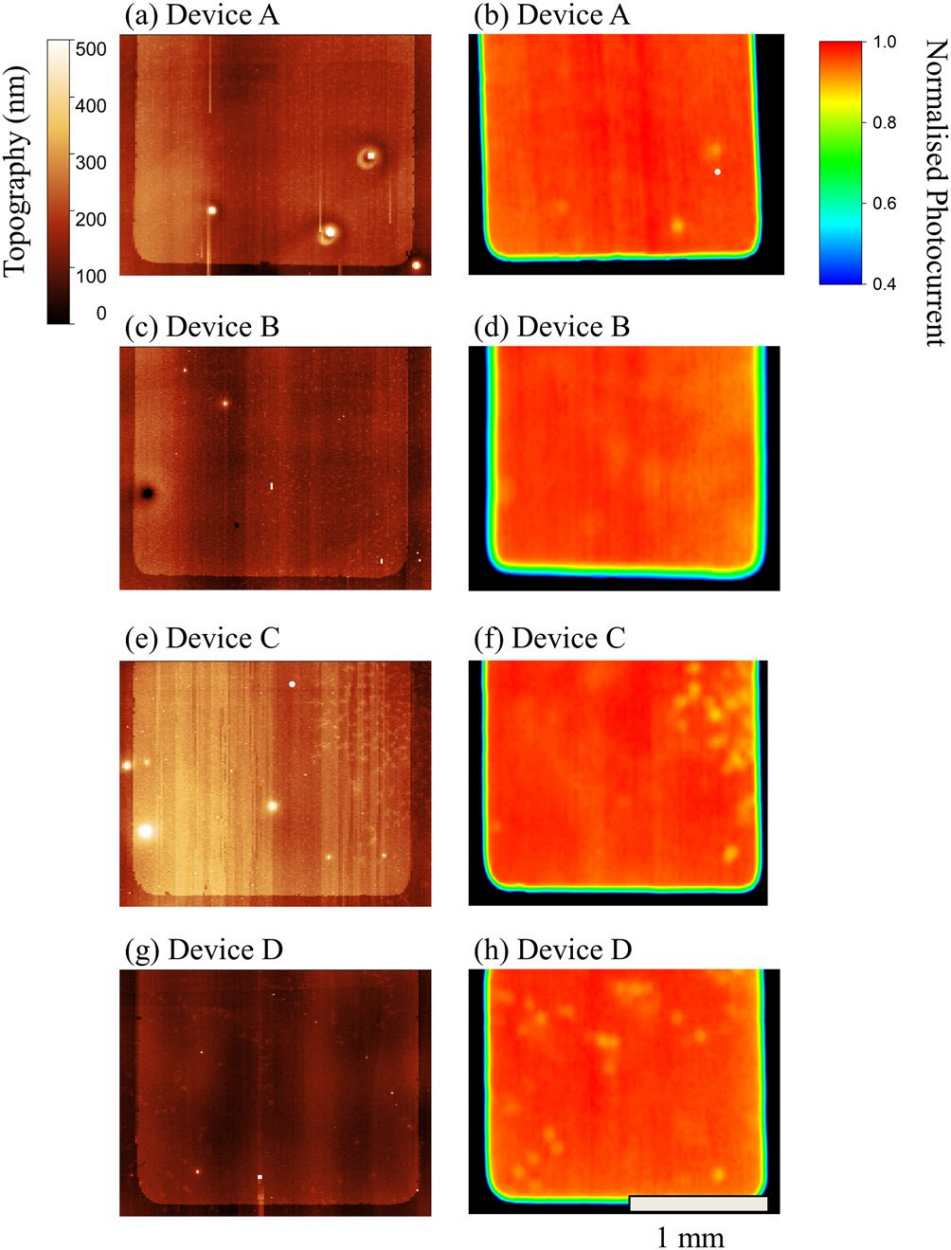
Topographical (panels a,c,e,g) and laser-beam-induced current mapping (panels b,d,f,h) images of spray-coated perovskite solar cells. See Table 1 for a description of device labels.

We have performed profilometry and laser-beam-induced current mapping (LBIC) on a series of typical devices fabricated by spin and spray-coating. Here, a Dektak surface profilometer was used to record a topographical image of the device surface. The same device was then scanned by a 25 µm laser spot whilst recording the photocurrent, allowing the photovoltaic response of device to be mapped. The results of these measurements are shown in Fig. 3.

As more layers are sprayed, the photocurrent uniformity remains high (see Fig. S5); a finding that accounts for the consistent values in PCE determined from the different device sets. However we do occasionally observe localised areas of reduced photocurrent. For example, Device A contained three regions of reduced photocurrent having a diameter of around 100 µm (so-called “cold spots”) that seem to be correlated with morphological defects in the topographical map. We have observed such defects before^20,29^ and suspect that they result from aggregates in the perovskite layer. Indeed, we also observe similar defects in the topographical images recorded from the other devices, indicating that it is likely that they are present in the perovskite film. Interestingly however, some topographical asperities do not appear to lead to a reduction in the photocurrent, with some current cold-spots being apparently uncorrelated with obvious morphological defects. The origins behind such effects are still not well understood.

On spray-coating the spiro-OMeTAD layer, we observe a small increase in the density of regions associated with lower average photocurrent. These seem to correlate to thickness fluctuations of the order of tens of nanometres, and occur over lateral length-scales of hundreds of microns in both Devices C and D. We speculate that these are likely correlated with fluctuations in the thickness of the spiro-OMeTAD layer caused as a result of a non-uniform drying process. Indeed, it appears that thicker parts of the spiro-OMeTAD layer result in a localised increase in series resistance and cause a small drop in photocurrent.

### Large-area devices

**Figure 4:**
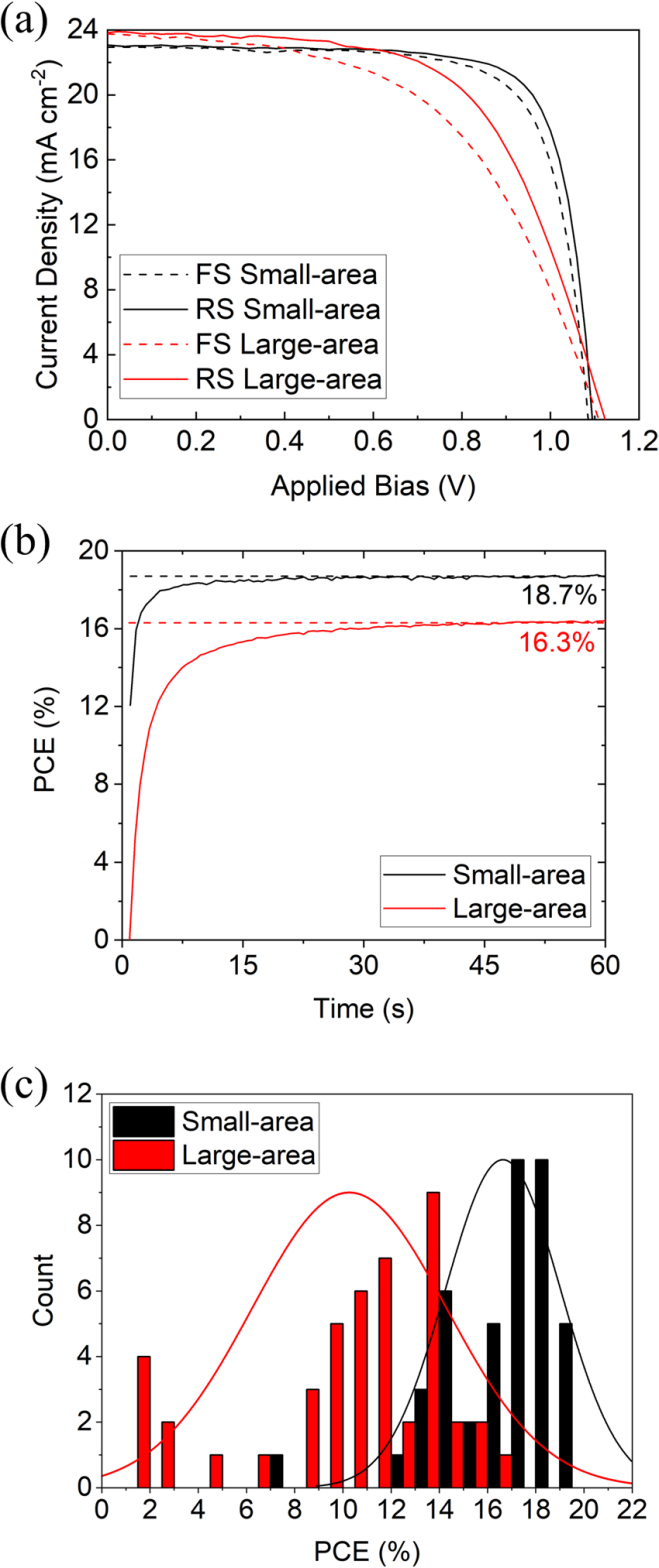
(**a**) Current-voltage characteristics for “champion” fully spray-cast perovskite solar cells with an active area of 2.5 mm^2^ (small-area) and 15.4 mm^2^ (large-area). The reverse scan PCE for these sweeps are 19.4% and 16.3% for the small and large-area cells respectively. (**b**) Output power of the champion devices when held (for 60 s) at a fixed voltage close to the maximum power point, indicating a stabilised PCE of 18.7% and 16.3% for small and large-area cells respectively. (**c**) A histogram of reverse-scan PCE data from 43 fully spray-cast small-area devices and 45 large-area devices.

We have used our spray-coating process to perform a limited scale up of device area. Here, four large-area substrates were coated that comprised a total of 48 device pixels, with each device having an active area of 15.4 mm^2^. The “champion” fully spray-coated large-area device had a reverse scan PCE of 16.3% relative to 19.4% recorded on small-area substrates (see Fig. 4(a)). Figure 4(b) shows the stabilised efficiency from the champion cells having a PCE of 18.7% and 16.3% for small and large-area cells respectively. A PCE histogram of these devices is shown in Fig. 4(c) where the small-area cells have an average PCE of 16.6 ± 2.4% while the large-area cells have an average PCE of 10.3 ± 4.0%. The other average performance metrics for such large-area devices are shown in Table 1 along with those of the champion device. Here it is clear the average performance of the 45 functioning large-area devices is reduced by seven devices that had an efficiency below 5% due to the presence of defects within their active area. In addition we note that the VASP treated large-area perovskite films are slightly rougher than those deposited on small-area substrates likely accounting for the reduction in PCE we observe. We anticipate that further optimisation of the VASP process can mitigate this effect.

This variability in performance between devices would clearly reduce the efficiency of a module in which all devices were connecting in series. However by parallel connecting 7 of the highest performing devices on one particular substrate, we were able to create a device having an effective area of 1.08 cm^2^ (see Fig. S6) having a reverse scan PCE of 12.7%. This efficiency is significantly greater than that reported in our previous study in which we fabricated a fully spray-cast device having an active area of 1.008 cm^2^ and PCE of 6.6%^29^. These results suggest a promising degree of uniformity across the entire coating area (approximately 18.8 cm^2^) although future work will involve further development of the deposition conditions to minimise the defect density. Here the use of other techniques such as *in-situ* Raman and local absorption spectroscopy could be utilised to further understand the composition of such defects. We believe that improved control of defect density should allow the fabrication of fully spray-cast mini-modules having a PCE of ~16%.

## Discussion

We have developed a process to fabricate perovskite solar cells in which all three solution processable layers were deposited via spray-coating. Using this process, we have been able to fabricate the most efficient spray-coated PSCs reported to date with a reverse scan PCE of 19.4% on small-area substrates. Furthermore, the spray-method developed allowed us to coat a relatively large area substrate (25 mm × 75 mm) at speed, with twelve 15.4 mm^2^ devices produced per substrate. These devices had a champion PCE of 16.3% and an average PCE of 10.3 ± 4.0%. By simultaneously connecting seven of these devices in parallel, we created a device having a PCE of 12.7% with an active-area of 1.08 cm^2^. We have characterised the quality of spray-cast devices using a combination of surface profilometry and laser-beam-induced current mapping, and find that device performance is reduced by the presence of aggregates and voids within the perovskite, as well as possible thickness fluctuations in the spiro-OMeTAD layer. In order to further improve device reproducibility and performance, it will be necessary to determine the precise origin of these morphological defects in the various device layers and develop strategies to reduce their areal density. Nevertheless, this work demonstrates a fast, scalable process by which efficient perovskite solar cells can be fabricated.

## Methods

### Device fabrication

FAI (Ossila), MABr (Dyesol), PbBr_2_ (TCI), PbI_2_ (TCI) and CsI (Sigma) were weighed out into a vial to form a triple cation perovskite with composition Cs_0.05_FA_0.81_MA_0.14_PbI_2.55_Br_0.45_. For each 1 mL of precursor solution, the following quantities of powder were used: FAI (167 mg), PbI_2_ (467 mg), MABr (19 mg), PbBr_2_ (68 mg) and CsI (16 mg). The powders were then dissolved in a mixture of DMF and DMSO at a ratio of 4:1 (800 µL and 200 µL) to form the perovskite precursor solution.

Small-area devices were fabricated on 15 × 20 mm unpatterned ITO substrates (20 Ω/sq, Ossila) which were etched with 4 M HCl and zinc powder. Large-area devices were fabricated on pre-patterned 25 × 75 mm ITO substrates (Ossila). Prior to deposition, substrates were cleaned via sonication in Hellmanex, deionised water and IPA. The substrates were then treated with a UV ozone cleaner for 15 minutes.

For spin coating, tin oxide nanoparticle solution (SnO_2_ colloidal solution 15% wt water) was diluted at 1:4 in DI water and spin coated under ambient conditions onto the ITO at 3000 rpm. The tin oxide was then heated for 30 minutes at 150 °C and UV ozone treated for a further 15 minutes.

For spray-coating, np-SnO_2_ solution was diluted 1:70 in DI water and spray-cast in air using a Prism Ultra-coat 300 system. The spray-head was programmed to move across the substrate at a speed of 180 mm s^−1^ at a height of 30 mm, coating a substrate held at 30 °C in a single pass. The flow rate was determined via the nitrogen feed into the fluid reservoir which was set to a pressure of 10 mbar. After 45 s the film had dried and the tin oxide layer was heated for 30 minutes at 150 °C and then UV ozone treated for a further 15 minutes.

The SnO_2_ coated substrates were then transferred to a glovebox for spray deposition using a Sonotek Exactacoat system mounted with an “Impact” spray-head. The perovskite precursor was delivered at 1 mL min^−1^ to the surface through a tip driven at 2 W using a N_2_ shaping gas at 3 Psi. The head was held 10 cm above the substrate which was mounted on a hotplate held at 40 °C. During deposition, the head moved in a line scan over the substrate at 80 mm s^−1^. The width of the spray pattern was around 5 cm, allowing the coating of both the small and large-area substrates in a single pass.

After deposition, the substrate was left for 30 s to allow an even wet film to form. The substrate was then transferred to the glovebox antechamber for vacuum exposure. The film was left for 1 minute in the vacuum chamber whilst it pumped down to approximately 80 Pa. After 1 minute, the vacuum chamber was rapidly re-filled with nitrogen. The film was then returned to the glovebox and placed on a hotplate at 120 °C for 20 minutes.

Perovskite films were transferred to a second glovebox for spin coating the spiro-OMeTAD layer. Here, 2,2′,7,7′-Tetrakis[N,N-di(4-methoxyphenyl)amino]-9,9′-spirobifluorene (spiro-OMeTAD) powder was first dissolved in CB at a concentration of 86.6 mg mL^−1^. This was then doped with lithium bis(trifluoromethanesulfonyl)imide (LITFSI Sigma), 4-tert-butyl-pyridine (TBP Sigma), and tris(2-(1H-pyrazol-1-yl)-4-tert-butylpyridine)cobalt(II) di[hexafluorophosphate] (FK209 Co(II) PF6 Dyesol). The quantity of dopants added to 1 mL of spiro-OMeTAD solution was as follows: 20 µL of LiTFSI (500 mg mL^−1^ in acetonitrile), 34 µL TBP, and 11 µL of FK209 (300 mg mL^−1^ in acetonitrile). The solution was mixed and finally filtered before being spin coated at 4000 rpm.

For spray-coating, the doped and filtered spiro-OMeTAD solution was diluted to 14 mg mL^−1^ in a 1:1 mixture of CB and CF. This solution was then spray-coated in air using the Prism Ultra-coat 300 system. The spray-head was programmed to move across the substrate at a speed of 150 mm s^−1^ and a height of 60 mm in a single pass over the substrate, which was held at 30 °C. The flow rate was defined by the nitrogen pressure which was set to 20 mbar. See Table 2 for a summary of all spray-deposition parameters.

**Table 2:** Summary of spray parameters used to fabricate perovskite solar cells.

Spray Parameter	np-SnO_2_	Perovskite	Spiro-OMeTAD
Spray-coater	Prism Ultracoat 300 Ambient Lab Conditions	Sonotek Exactacoat, Impact Spray-Head, Glovebox	Prism Ultracoat 300 Ambient Lab Conditions
Substrate Temp (°C)	30	40	30
Head Height (mm)	30	100	60
Head Velocity (mm s^−1^)	180	80	150
Flow Rate (mL min^−1^)	N/A	1	N/A
Fluid Pressure (mbar)	10	N/A	20

After the deposition of spiro-OMeTAD, films were left overnight in dry air to oxidise. A 100 nm thick gold film was then deposited through an evaporation mask to pattern individual cell areas, at a pressure of ≈10^−6^ mbar in an Edwards bell jar evaporator. Small-area devices were mounted in a mask that defined six (2 × 2) mm cells per substrate. Large-area devices were patterned through a mask defining twelve (10 × 2) mm cells per substrate.

### Current-voltage measurements

Devices were tested under AM 1.5 illumination using a Newport Solar Simulator. The light intensity was calibrated to 1000 Wm^−2^ using a silicon reference cell (Newport). Devices were swept from −0.2 V to 1.2 V and back to −0.2 V at a scan rate of 0.4 Vs^−1^ using a Keithley 237 source measure unit. Small-area and large-area devices were tested through illumination masks having an area of 2.5 mm^2^ and 15.4 mm^2^ respectively. By measuring several devices over the large-area substrates in parallel, the performance of larger active areas could be established. For such measurements, a slower scan rate of 0.1 Vs^−1^ was employed. Stabilised measurements were recorded by holding the device at a point close to the maximum power point for 60 s whilst reading the current.

### External quantum efficiency

EQE measurements were performed using a custom setup. Light from a 100 W tungsten halogen lamp was passed through a monochromator (Spectral Products DK240 1/4 m) and then focussed onto the device. Photocurrent was measured using an Xtralien X100 source measure unit (Ossila) and compared to the current produced by a silicon reference photodiode (Newport) with a known spectral response that was used to calculate the EQE.

### Surface profilometry and laser-beam-induced current mapping

A Bruker DektakXT was utilised to generate surface topography maps of perovskite solar cells (12.5 µm tip radius, 3 mg stylus force) over an area of (2 × 3) mm. Each image was generated from a series of 200 line scans separated by 15 µm, where each line scan covered a lateral distance of 2000 µm with a resolution of 0.333 µm per point.

The laser-beam-inducedcurrent (LBIC) mapping system comprised of a mechanically chopped laser that was passed through a spatial filter before being focused to a spot size of around 25 μm onto a device via a 10x objective. The sample was mounted on a computer controlled XY-stage, and moved in a sawtooth pattern in steps of 25 μm. A 1.2 mW, 632 nm laser (Thor labs, HRS015B) was used to generate a photocurrent that was measured using a lock-in amplifier (Stanford Research Systems, SR830) and referenced to the chopped laser.

### Scanning electron microscopy

Cross-sectional scanning electron microscopy (SEM) images were collected using a Carl Zeiss modified Raith Nanofabrication SEM working at a 1.5 kV accelerating voltage and ~2 mm working distance. The signal was gathered using an “InLens” detector with rapid acquisition on image areas to minimise sample beam damage.

## Supplementary information

Supplementary Information.
