# Supplementary material for: Fully Spray-Coated Triple-Cation Perovskite Solar Cells
Source: Sci Rep. 2020 Apr 20;10:6610. doi: 10.1038/s41598-020-63674-5 (PMC7171188; doi:10.1038/s41598-020-63674-5)
Supplement: Supplementary file 1 — Supplementary Information. [file 41598_2020_63674_MOESM1_ESM.pdf]

Fully Spray-Coated Triple-Cation Perovskite Solar Cells

James E. Bishop, Connor D. Read, Joel A. Smith, Thomas J. Routledge, David G. Lidzey*

Department of Physics & Astronomy, University of Sheffield, Hicks Building, Hounsfield Road, Sheffield, S3 7RH, U.K.

*Corresponding author, email d.g.lidzey@sheffield.ac.uk

Supplementary Information

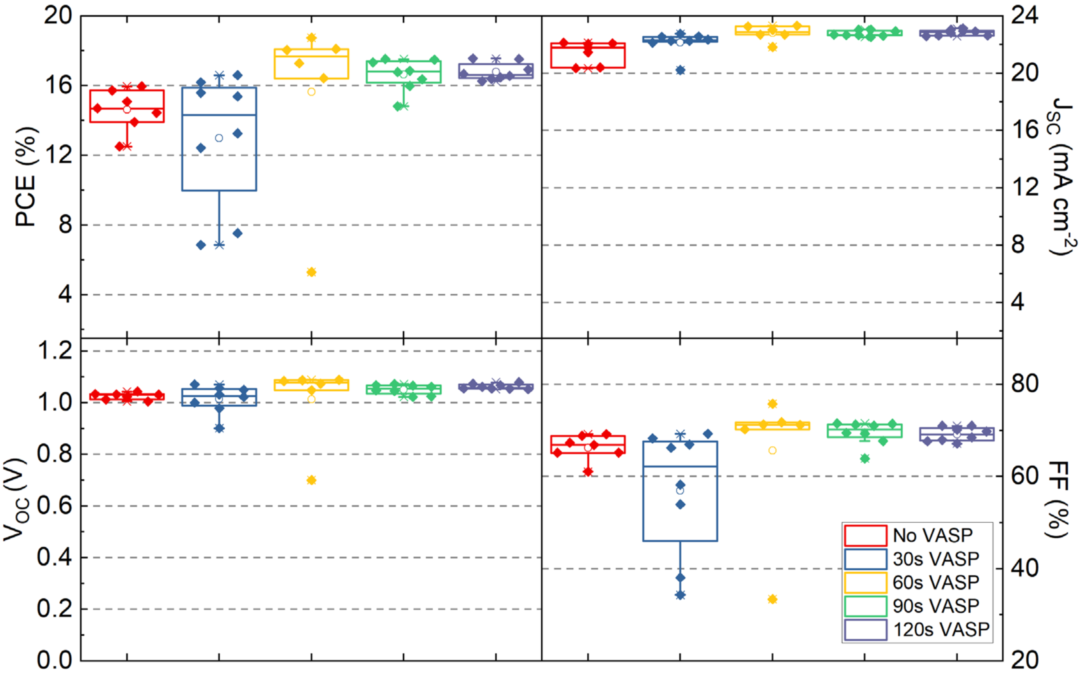

Figure S1: *Effect of varying vacuum exposure time on device performance of small-area spray-cast PSCs. Here it can be seen that a minimum of 1 minute of exposure is required for high performance.*

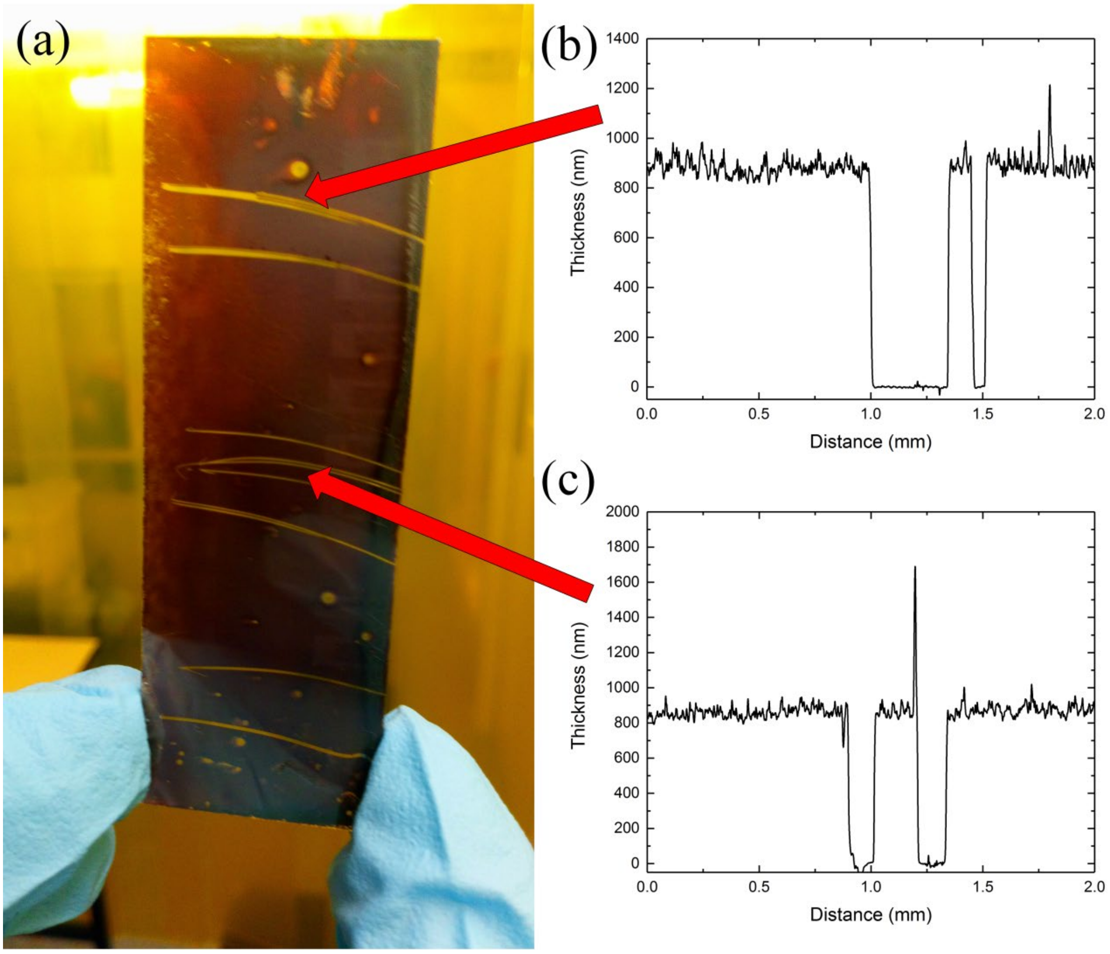

Figure S2: *(a) Photograph of a spray-coated perovskite film. Here the presence of several circular voids in the film is apparent as well as scratches made in the film for thickness measurements. (b) and (c) show Dektak line scans used to estimate the thickness of the layer (850±100 nm).*

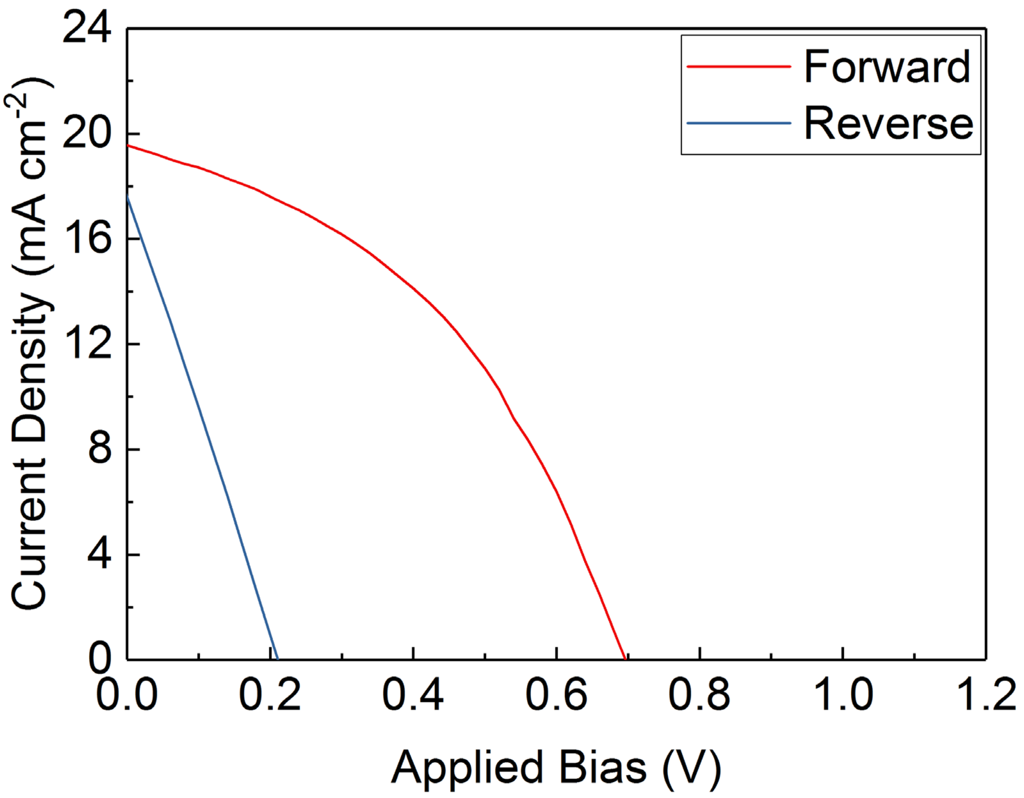

Figure S3: *Example of a "failed" device where the reverse scan PCE is less than 1%.*

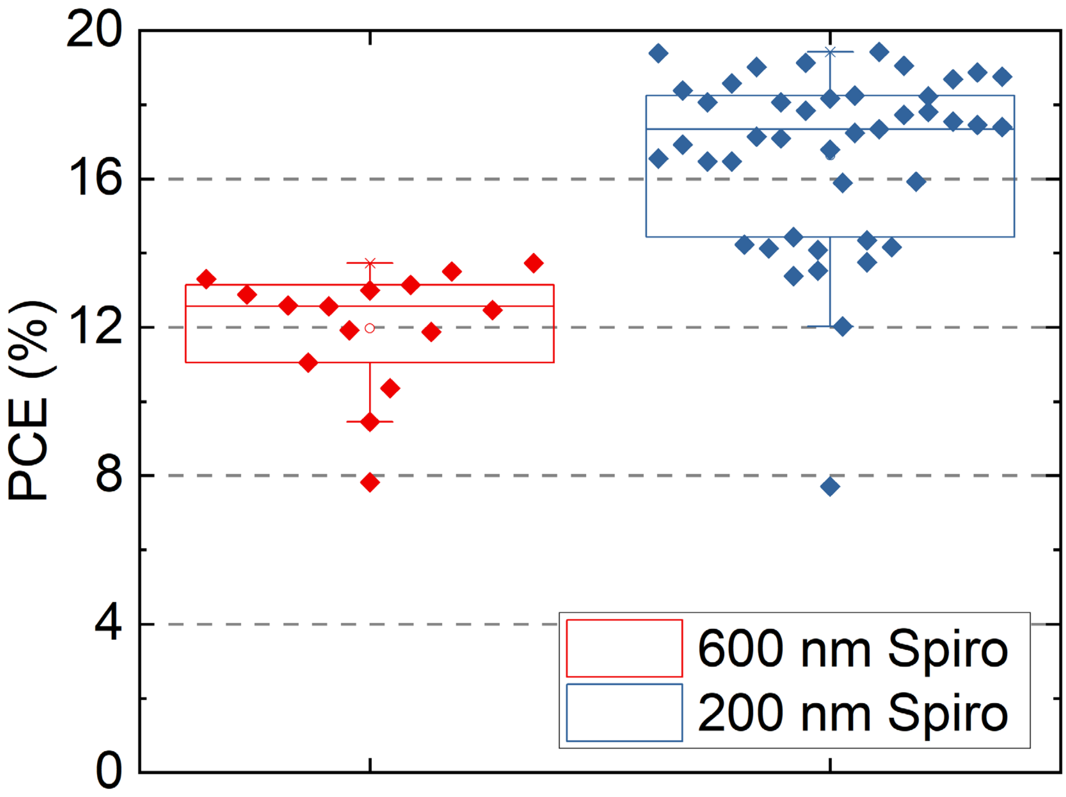

Figure S4: *PCE box plots for small-area fully spray-coated PSCs where the spiro-OMeTAD was sprayed from solutions with a concentration of 43 mg/mL and 14 mg/mL (all other spray parameters were fixed). When spraying from the lower concentration solution the thickness of the sprayed layer reduces from 600 nm to 200 nm. This results in improved performance.*

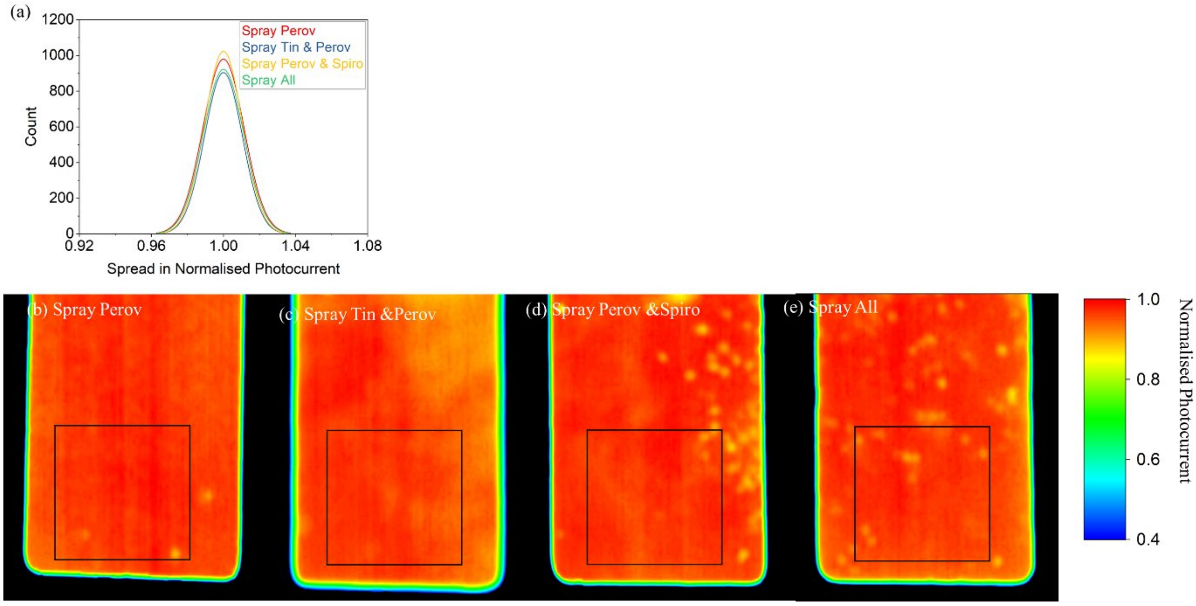

Figure S5*: (a) Histograms showing the spread in normalised photocurrent around the mean value for various sprayed PSCs taken from LBIC measurements. (b) to (e) show the LBIC images where the black boxes denote the areas sampled to create the histograms shown in (a). Here it is clear that as more layers are sprayed the spread in photocurrent values remains approximately constant, which accounts for the limited reduction in device PCE.*

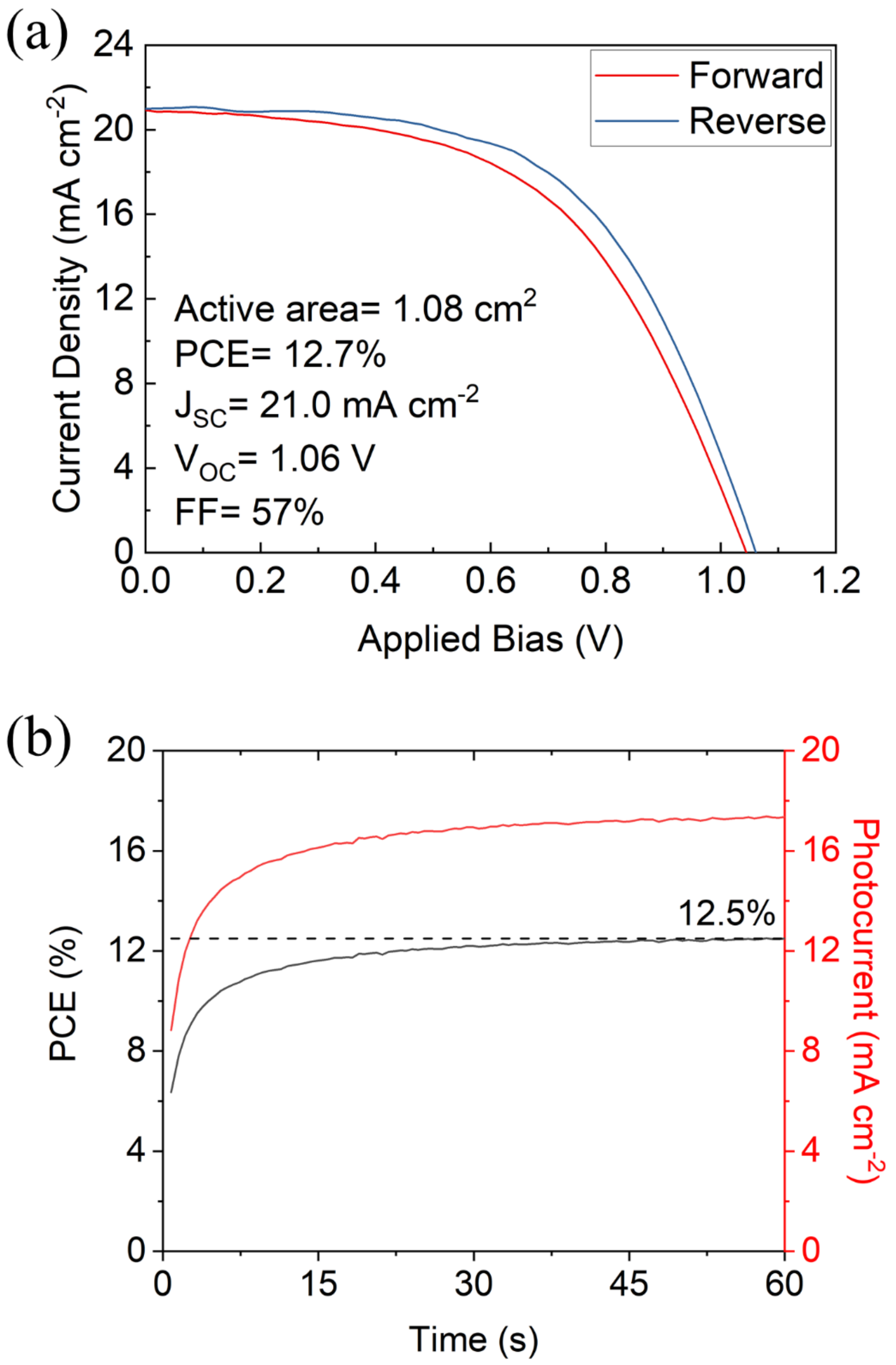

***Figure S6**: (a) Current-voltage characteristics for seven 15.4 mm^2 devices on one large-area substrate connected in parallel, creating a device with an effective active-area of 1.08 cm^2. (b) Stabilised power output for the same ten parallel connected devices held at 0.72 V for 60 s. The effective power output for this device is 13.5 mW.*

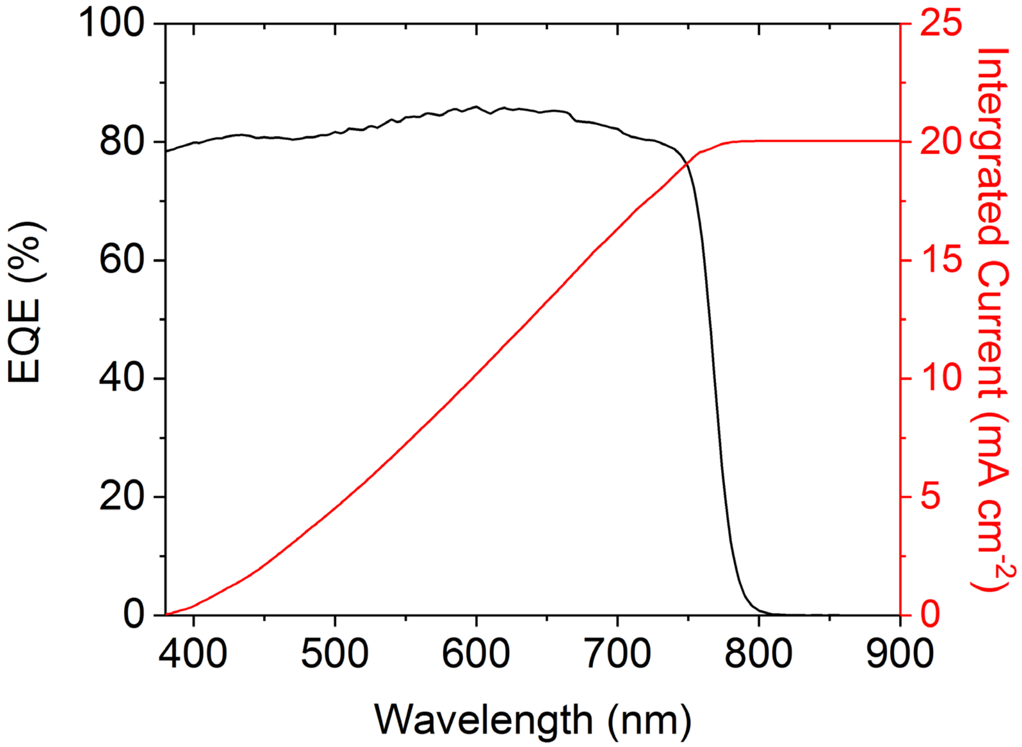

***Figure S7**: EQE spectrum for a fully spray-coated perovskite solar cell showing an integrated J_{SC} of 20 mAcm^{-2}. Note due to technical limitations with our EQE setup we are unable to measure spectral response below 380 nm thus the integrated J_{SC} is likely to be a slight underestimate for this particular device.*
